# COVID-19 vaccines and neurological disorders: A narrative review of immune responses and adverse reactions

**DOI:** 10.3934/Neuroscience.2025013

**Published:** 2025-06-18

**Authors:** Mehran Joodaki, Farhad Seif, Azadeh Afzalnia, Nikoo Emtiazi, Mona Merati Shirazi, Behnaz Ashtari, Seyed Mohamad Hosseinian, Nasrin Hosseini

**Affiliations:** 1 Shariati Hospital, Tehran University of Medical Sciences, Tehran, Iran; 2 Department of Photodynamic, Medical Laser Research Center, Yara Institute, Academic Center for Education, Culture, and Research (ACECR), Tehran, Iran; 3 Rajaei Cardiovascular Medical and Research Center, Iran University of Medical Sciences, Tehran, Iran; 4 Department of Pathology Medicine, Rasool Akram Hospital, School of Medicine, Iran University of Medical Sciences, Tehran, Iran; 5 Biomedical Engineering Faculty, Biomechanics Department, Islamic Azad University, Science and Research Branch, Tehran, Iran; 6 Radiation Biology Research Center, Iran University of Medical Sciences, Tehran, Iran; 7 Department of Medical Nanotechnology, Faculty of Advanced Technologies in Medicine, Iran University of Medical Sciences, Tehran, Iran; 8 Rheumatology Department, Shafa Hospital, Kerman University of Medical Sciences, Kerman, Iran; 9 Neuroscience Research Center, Iran University of Medical Sciences, Tehran, Iran

**Keywords:** immunogenicity, COVID-19, vaccine, neurological complication, neurodegeneration

## Abstract

COVID-19 vaccines have been effective in providing strong immunity within a relatively short time frame, significantly reducing both the severity of the disease and associated mortality. However, post-vaccination complications, particularly neurological disorders, have been reported. Among the more frequently documented neurological complications are acute disseminated encephalomyelitis (ADEM), multiple sclerosis (MS), transverse myelitis (TM), optic neuritis (ON), Bell's palsy (BP), and Guillain–Barré syndrome (GBS). The precise role of COVID-19 vaccines in triggering the onset or relapse of these conditions remains uncertain. Immunological processes involving cytokines, chemokines, antibodies, and immune cells are believed to contribute to the pathogenesis of these neurological side effects. This review examines the immune responses triggered by COVID-19 vaccines and their potential role in the development of such complications. Despite reports of neurological side effects, these cases remain rare, and the overall benefits of vaccination in curbing the pandemic and preventing severe illness far exceed the risks. It is vital to sustain the progress of global vaccination efforts while continuously evaluating the risk-benefit ratio, particularly for individuals with underlying conditions. Ongoing research and surveillance are crucial for creating safer vaccines and identifying individuals who may be more susceptible to adverse reactions, ensuring broader protection against COVID-19.

## 1. Introduction

The global outbreak of SARS-CoV-2 spurred an urgent worldwide effort among researchers to develop a vaccine. Efforts resulted in the production of a vaccine based on the spike protein [1]. Following the initial vaccine development, the Pfizer-BioNTech (BNT162b2), AstraZeneca (Covishield, Vaxzevria, ChAdOx1-S/n, AZD1222, AZD2816), and Moderna (mRNA-1273) vaccines were released on December 31, 2020, February 15, March 12, and April 30, 2021, respectively. After clinical trials evaluating their efficacy and safety, Pfizer-BioNTech, Moderna, Janssen/Johnson & Johnson (Ad26.COV2.S), Sputnik V (Gam-COVID-Vac/AZD2816), and SinoVac (CoronaVac) vaccines were also granted approval for use [2]. The developed vaccines are categorized into 11 main platforms (Supplementary Table S1) [1]. Additional details about the types and structures of these vaccines are summarized in Table 1. The mRNA vaccines, including those developed by Pfizer-BioNTech and Moderna, utilize messenger RNA to direct host cells to synthesize the spike protein, which subsequently stimulates an immune response. In contrast, vector-based vaccines, like AstraZeneca and Janssen, employ a modified adenovirus to deliver the genetic material for the spike protein. As of March 30, 2023, the WHO has listed over 382 vaccines, with 199 in preclinical stages and 183 undergoing clinical trials [3]. The rapid development and approval of these vaccines have dramatically improved global vaccine coverage, resulting in a significant reduction in hospitalizations and deaths, particularly in countries with extensive vaccination campaigns.

**Table 1.** Different types of vaccines and structures.

**Vaccine type**	**Names**	**Structures**	**Ref**
**DNA vaccines**	INO–4800 (Inovio/International Vaccine Institute)	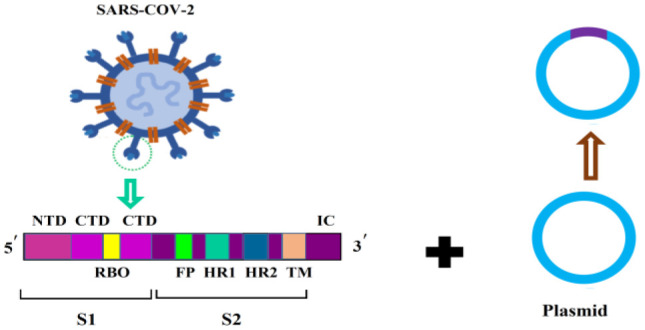	[4]
**mRNA vaccines**	Moderna's vaccine (mRNA–1273) BNT162b2 (Pfizer/BioNTech/Fosun Pharma) CVnCoV (CureVac)	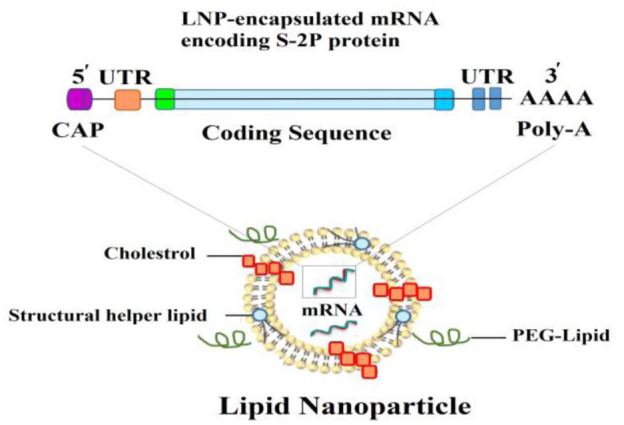	[5]
**Non-replicating viral vector vaccines**	Janssen/“Johnson & Johnson vaccine” (Jcovden/Ad26.COV2.S) JNJ-78436735/Ad26.COV2.S (Janssen and Beth Israel Deaconess Medical Center) Oxford and AstraZeneca ChAdOx1/AZD1222) Ad5-nCOV (Convidecia)	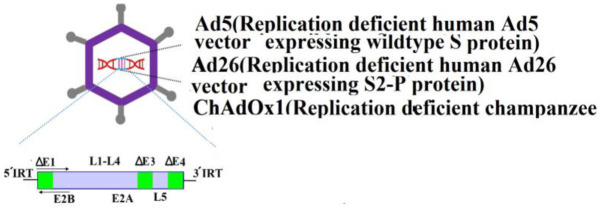	[6]
**Inactivated vaccines**	Inactivated SARS-CoV–2 vaccine (Vero cells) BBIBP-CorV vaccine (Sinopharm) Sinovac (CoronaVac/PiCoVacc) Covaxin/BBV152 (Bharat Biotech/Indian Council of Medical Research/National Institute of Virology	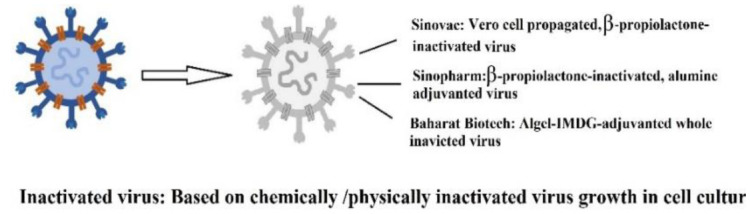	[7]
**Live attenuated vaccines**	Polio vaccine Adenovirus vaccine (Sputnik V/ (Gam-COVID-Vac)/Gamaleya) NVX-oV2373(Nuvaxovid/Covovax) CoVLP (*Covifenz*) (Medicago)	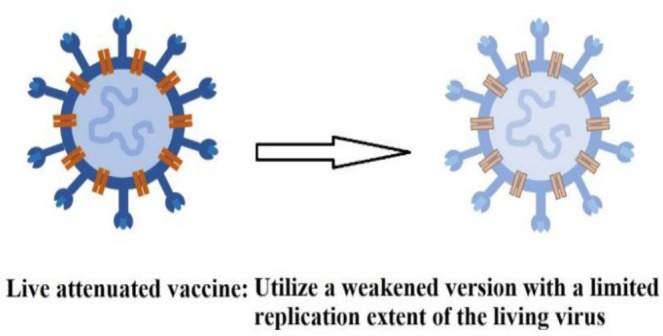	[8]
**Protein subunit vaccines**	NVX-CoV2373 (Novavax) ZF2001(Zifivax or ZF-UZ-VAC-2001:(Anhui Zhifei Longcom Biopharmaceutical/Chinese Academy of Medical Sciences) Unknown name (Sanofi Pasteur/GlaxoSmithKline)	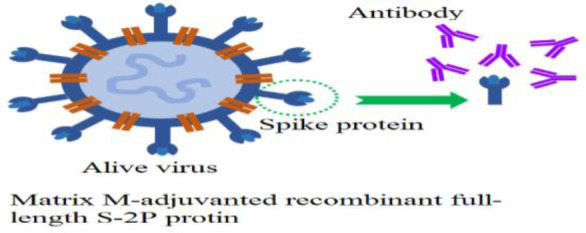	[9]
	Recombinant subunit SARS-CoV–2 vaccine (CHO cells)	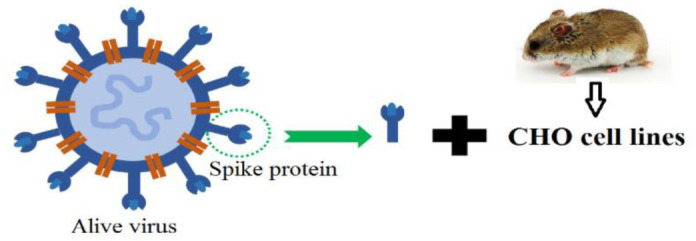	[10]
**Trained immunity-based vaccines**	BCG vaccine	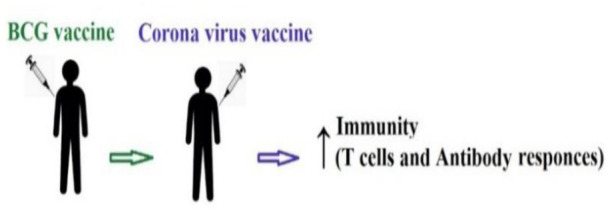	[11]

Although vaccines are essential in stimulating the immune system to target specific antigens, some local, systemic, and central side effects are unavoidable. Neurological complications, including headaches, anosmia, paresthesia, dizziness, seizures, cranial nerve palsies, peripheral myopathy or neuropathy, encephalopathy, and stroke, have been documented following either SARS-CoV-2 infection or vaccination [12]–[14]. Although severe neurological complications have been rarely reported in a small percentage of vaccinated individuals, the benefits of vaccination in preventing severe disease and mortality from COVID-19 significantly outweigh the adverse event risks. Accordingly, public health authorities continue to emphasize that these incidents should not discourage vaccination. The range and frequency of neurological complications also vary among vaccines, affecting both genders, with differences observed in dosage and the timing of onset post-vaccination [15]. Headaches have been observed following the administration of the initial dose of Moderna, Sinopharm, AstraZeneca, and Sputnik V vaccines. Furthermore, some cases of demyelinating diseases, neurological disorders, and cerebral venous sinus thrombosis have been observed after vaccination with Moderna, Pfizer-BioNTech, and Janssen [16],[17]. Neurological complications, e.g., stroke, encephalopathy, and GBS are much more likely to occur from severe COVID-19, which can also lead to long-term health issues or even death. Thus, vaccination remains one of the most effective strategies for mitigating these risks.

In the past, GBS, MS, myelitis, and other demyelinating disorders have been reported following vaccinations for influenza, polio, hepatitis B, typhoid fever, rabies, tetanus, smallpox, and tuberculosis. More recently, an association between acute demyelinating diseases and COVID-19 (occurring 1–7 days and 8–14 days post-infection), as well as vaccination (particularly with AstraZeneca), have also been observed. Some studies reported a case of both peripheral and central nervous system (CNS) demyelination, marked by deep sensory impairment extending to the clavicles and multiple subcortical and periventricular lesions visible on MRI following vaccination [18]. However, other studies revealed no correlation between COVID-19 vaccination and acute CNS demyelination [19]. These conflicting reports have contributed to vaccine hesitancy among some individuals. Some studies reported that the incidence of neurological complications during the COVID-19 pandemic was up to 617 times greater than that observed following COVID-19 vaccination [20]. The COVID-19 pandemic has posed major challenges to the treatment of neurological patients, with varied responses and resource availability across and within countries [21]. In this review, we discuss the immunological mechanisms involved in immune-related events affecting the nervous system following SARS-CoV-2 infection and/or vaccination. Moreover, we provide broader insights into the correlation between COVID-19 vaccines and neurological complications.

## 2. Immunogenicity of SARS-CoV-2 infection

SARS-CoV-2 enters the body through the naso-oral cavity, binding to angiotensin-converting enzyme 2 receptor (ACE2) receptors on the upper respiratory tract epithelial cells, where it commences an asymptomatic replication phase [22]. During the latent phase, innate immune cells remain quiescent. However, once the infection progresses to the lower respiratory tract, it can provoke a severe innate immune response, leading to elevated production of pro-inflammatory cytokines (cytokine storm), potentially causing pulmonary edema, acute respiratory distress syndrome (ARDS), and even death [23],[24]. Pathogen recognition receptors (PRRs), including toll-like receptors (TLR) 3, 7, and 8 on immune cells, play a key role in detecting the virus and initiating the production of type I interferons (IFNs) [25],[26]. Notably, certain risk factors, such as advanced age, pre-existing comorbidities (e.g., cardiovascular disease, diabetes, or autoimmune disorders), and genetic predispositions, may make individuals more susceptible to severe outcomes or adverse reactions. These factors can escalate the immune response and increase the likelihood of neurological complications or other systemic issues.

In contrast, the humoral immune response to a viral infection triggers the synthesis of IgM, IgG, and IgA antibodies. IgM and IgA antibodies are generally detectable about 5 days after the onset of symptoms, while IgG antibodies typically appear around 14 days later [27]. IgM appears early in infection, signaling recent exposure and offering short-term protection, while IgG develops later to provide long-term immunity by neutralizing the virus and preventing reinfection. This differentiation is pivotal to understanding how the immune system mounts both rapid and lasting defenses against viruses over time. During the acute phase of infection, the nucleocapsid (N) proteins of the virus are recognized by B cells or presented to CD8+ and CD4+ T cells via major histocompatibility complex (MHC) class I and II molecules, respectively, thereby increasing antibody production, cytokine secretion, and cytolytic activity [28]. The spike (S) protein serves as a key immunodominant epitope for the activation of CD8+ T cells in SARS-CoV infection [29]. A laboratory analysis of 20 individuals with confirmed COVID-19 revealed that T-helper cells generated a robust adaptive immune response targeting the N, S, and M proteins of the virus. This was accompanied by strong CD4+ and CD8+ T-cell activity, along with elevated levels of anti-S-RBD-specific IgG and IgA antibodies. Notably, similar T-cell responses were observed in 40%–60% of uninfected individuals previously exposed to other coronaviruses, suggesting that prior exposure could lead to T-cell exhaustion during severe viral infections [30]. Furthermore, the ACE2-independent pathway in Fc receptor (FcR)-expressing cells facilitates viral spread without replication, a phenomenon known as the non-neutralizing antibody mechanism. During this mechanism, macrophages engage with the virus, initiating inflammatory cascades and inducing tissue injury via the activation of myeloid cell populations. This can lead to abnormal immune cell activation, a process termed antibody-dependent enhancement (ADE) [31]. While ADE has not been documented in SARS-CoV-2 infections, it remains crucial to consider this potential risk when developing vaccines and antibody-based therapies [32],[33].

Cytokines and chemokines play a critical role in both innate and adaptive immune responses. SARS-CoV-2 infection is associated with elevated levels of cytokines and chemokines, including interleukin-6 (IL-6), tumor necrosis factor-alpha (TNF-α), macrophage inflammatory protein 1-α (MIP-1α), monocyte chemoattractant protein-3 (MCP3), granulocyte-macrophage colony-stimulating factor (GM-CSF), CCL2/MCP1, CXCL1, CXCL5, IL-2, and interferon-γ-induced protein 10 (IP-10) [28]. In the innate immune response, activated macrophages and natural killer (NK) cells produce cytokines such as IL-2, IL-6, IL-17, TNF-α, interferon-gamma (IFN-γ), MIP-1α, and IP-10. CD4+ T cells release a range of cytokines to regulate immune functions, including IFN-γ (Th1 cells), IL-4, IL-5, IL-10, and IL-13 (Th2 cells), IL-17 and IL-21 (Th17 cells), and transforming growth factor-beta (TGF-β) (Treg cells), contributing to both cellular and humoral immune responses [28],[34],[35].

## 3. Immune response to COVID-19 vaccines

All vaccines stimulate the immune system to generate immune responses. Inactivated vaccines predominantly induce a humoral response and typically require multiple doses to achieve protective immunity. In contrast, live-attenuated vaccines elicit both a stronger cellular and humoral response with fewer doses. However, vaccines can occasionally cause adverse side effects, particularly in individuals with immunodeficiency, chronic illnesses, or those who have undergone organ transplants. Vaccine antigens or adjuvants, such as aluminum salts, can also stimulate immune responses and lead to the production of specific self-antigen antibodies [36]. To address this, researchers have worked to eliminate the use of adjuvants, opting instead for lipid nanoparticles to deliver the genetic material encoding the viral S-protein in the new mRNA vaccines. Lipid nanoparticles provide several benefits, such as improving the stability of mRNA and enabling targeted delivery to cells, thereby increasing the vaccine's efficiency. These nanoparticles protect mRNA from degradation and facilitate its delivery into cells, where it triggers an immune response, ultimately making the vaccines more effective and durable in generating long-lasting immunity [37].

Upregulation of cytokine signaling genes and neutrophil degranulation have been observed following Moderna and Pfizer-BioNTech mRNA vaccinations [38]. The COVAXIN (BBV152) vaccine is an inactivated vaccine that demonstrates the ability to provoke a potent neutralizing antibody response while stimulating a heightened CD4+ Th1-mediated immune reaction [36]. Systemic symptoms following SARS-CoV-2 vaccination may be associated with cytokine storms. Studies have indicated that CXCL10 binding can activate the extracellular signal-regulated kinase (ERK) pathway, which may result in neuronal dysfunction and apoptosis [39]. Type I interferons are also involved in antigen-specific immune response. However, an unchecked and chronic IFN I-mediated innate immune response can result in lymphopenia, immature neutrophilia, and systemic inflammation [40]. It has been suggested that the overproduction of IL-10 and Th2 cytokines (IL-4, IL-5, and IL-13) may play a role in controlling cytokine storms [41]. In this context, trials have reported cytokine storms and the overproduction of IFN-γ, IL-2, IL-6, IL-15, IL-17, TNF-α, IP-10, CXCL10, and MIP-1α following Pfizer-BioNTech vaccination [42]. The first dose of the mRNA vaccine also triggered significant production of IL-8, IL-18, and MCP-1, accompanied by symptoms of perimyocarditis [43]. Additionally, cases of multisystem inflammatory syndrome and organ dysfunction have been documented after the initial administration of the Pfizer-BioNTech vaccine [44]. Assiri et al. (2022) reported neurological complications following the first dose of the AstraZeneca vaccine, which resulted in more severe neurological symptoms compared to the Pfizer-BioNTech vaccine. These complications may be associated with the release of TNF-α, IL-1β, and IL-6 from astrocytes and microglia [45].

Elevated levels of systemic pro-inflammatory cytokines can promote blood-brain barrier (BBB) disruption, alter immune cell phenotypes, and increase permeability. Similarly, elevated levels of IL-1β, IL-2, IL-6, IL-8, IL-15, IL-17, and IL-18 have been detected in cases of GBS, active MS, encephalitis, inflammatory myopathies, viral and bacterial meningitis, and stroke [46]–[51]. IP-10 levels are elevated in encephalitis, aseptic meningitis, and acute bacterial meningitis, while MIP-1α and MIP-1β are significantly increased in cases of myelopathy and MS [52],[53]. AstraZeneca (AZD1222), Covaxin, and Moderna vaccines have also been linked to nervous system complications, including seizures and brain strokes, which are associated with the production and release of spike protein through the vaccine-induced cellular machinery [54]. In brain strokes, inflammatory states may precipitate hypercoagulopathy, presenting as ischemic cerebral venous thrombosis (CVT) or stroke accompanied by intracranial hemorrhage. This triggers disseminated intravascular coagulation and vascular endothelial dysfunction, ultimately resulting in large-vessel stroke [45]. Inflammatory mediators may be directly released from the cerebral endothelial lining and cross a compromised BBB into the brain parenchyma. Inflammatory cytokines can disrupt cellular metabolism by activating microglia, leading to mitochondrial dysfunction and oxidative stress, which in turn cause neuropathological abnormalities, e.g., impaired neurotransmission and apoptosis [47]. Rapid and urgent spread of the coronavirus led to vaccines being introduced to the market without going through the standard FDA approval process. Now, after about 5 years, more detailed immune-pathophysiological mechanisms underlying the side effects of different COVID-19 vaccines are proposed.

## 4. Immunogenicity of COVID-19 vaccines with neurological complications

COVID-19 vaccine side effect databases have reported various neurological complications, ranging from mild to severe, affecting both local and systemic areas. Severe immune-mediated demyelinating conditions, including ADEM, MS, optic neuritis, neuromyelitis optica, TM, BP, and GBS, have been reported following both COVID-19 and vaccination. Although there is no strong evidence directly linking vaccines to an increased risk of demyelinating conditions, some vaccine types may affect the chances of neurological complications. Aberrant immune responses and molecular mimicry are proposed key mechanisms behind vaccine-related adverse effects and disease development. Seizures after COVID-19 vaccination may be linked to glial cell activation and BBB disruption, enabling immune cells and albumin to penetrate the brain. This infiltration disrupts osmotic homeostasis under hyperthermic conditions [45],[55]. Peripheral blood cells can secrete inflammatory mediators that penetrate the brain, causing myelin destruction and axonal degeneration. Fan et al. (2022) identified spike protein S1 antibodies in the cerebrospinal fluid (CSF) of individuals who developed encephalitis and status epilepticus following COVID-19 vaccination [56].

Patone et al. (2021) reported that the AZD122, Sinopharm/BIBP, and AstraZeneca vaccines caused CNS demyelination, even for a short duration, unlike the Pfizer-BioNTech or AstraZeneca vaccines [19]. Furthermore, Khayat-Khoei et al. reported that the SARS-CoV-2 mRNA vaccine resulted in positive MRI findings and clinical neurological symptoms of optic nerve, brain, and/or spinal cord demyelination a few weeks after the first or second dose. Additionally, two cases presented with new-onset demyelinating disease, while four cases experienced disease relapse [16]. Nevertheless, it is important to consider that the timing of vaccination and the onset of demyelination symptoms may be coincidental. The neurological side effects of vaccines are summarized in Table 2.

**Table 2.** Neurological complications reported for COVID-19 vaccines.

**Neurological adverse events**	**COVID vaccine name**	**Complications**
Stroke	AstraZeneca (ChAdOx1 n) Pfizer–BioNTech (BNT162b2, mRNA-1273), Moderna Janssen (Ad26.COV2-S), Sputnik V (Gam-COVID-Vac), CoronaVac (Ad5-nCoV), Sinopharm (BIBP)	Ischemic stroke, venous sinus thrombosis, intracerebral hemorrhage, transient ischemic attack, intracerebral hemorrhage
Encephalitis	Moderna (mRNA-1273), AstraZeneca (ChAdOx1-S), Pfizer–BioNTech (BNT162b2), Janssen	Paralysis of the facial nerve (weakness or complete loss of movement), cognitive decline, seizures, gait disorder
ON	Moderna, AstraZeneca (Vaxzevria), Pfizer–BioNTech (BNT162b2)	Vision loss, blurred vision, pain with eye movement, unilateral or bilateral optic nerve involvement
TM	AstraZeneca (Covishield), Moderna, Janssen, Pfizer–BioNTech (BNT162b2), and AstraZeneca (AZD1222)	Paresthesia and pain in the upper abdomen, abnormal sensations ascending the trunk (weakness and numbness), sphincter dysfunction, and balance issues
MS	Moderna, AstraZeneca (AZD1222, Covishield), Sputnik V (Gam-COVID-Vac), Pfizer BioNTech	Visual loss, visual symptoms (diplopia, blurred vision), pain with eye movement, gait instability, dysmetria, paresthesia in face and upper extremity, limb weakness, sphincter disturbances
ADEM	Moderna, AstraZeneca (AZD1222), Pfizer BioNTech, SinoVac (CoronaVac), Sinopharm (Vero Cells), Sputnik V (Gam-COVID-Vac), Covaxin (BBV152) Janssen (Ad26.COV2.S)	Visual impairments, blurred vision, quadriparesis, generalized weakness, myalgias, apathy, pain, paraplegic syndrome, muscle stiffness, unconsciousness, seizure, numbness and hypoesthesia, anosognosia, gait ataxia, fever, back pain, urinary retention, silent myocardial infarction, behavioral and memory disturbances
GBS	Moderna, AstraZeneca (AZD1222), Pfizer-BioNTech, Janssen (Ad26.COV2.S)	Bifacial weakness and distal paresthesia, numbness and quadriparesis, severe headaches in the temporal region, lower back and umbilical abdominal pain with paresthesia in hands and feet, bilateral proximal lower limb weakness as well as asymmetric facial weakness resulting in drooling and significant dysarthria
BP	Moderna, Pfizer-BioNTech, Janssen, CoronaVac, and Oxford-AstraZeneca	Facial paralysis, numbness, tingling, difficulty in speaking and eating, numbness and tingling of the left arm along with subjective weakness of the left upper limb, decreased sensations on the left upper limb that spared the lower limbs, loss of taste sensation along with tinnitus in the right ear, right-sided facial droop and loss of right naso-labial fold, as well as inability to close the right eye completely

Note: acute disseminated encephalomyelitis (ADEM); Bell's palsy (BP); Guillain–Barré syndrome (GBS); multiple sclerosis (MS), optic neuritis (ON); and transverse myelitis (TM).

## 5. Neurological complications

### 5.1. Stroke

Ischemic and hemorrhagic strokes are the most common types of neurological complications, while cerebrovascular venous sinus thrombosis (CVST) is less frequent. Stroke, defined as an acute neurological deficit due to CNS vascular injury, is the second leading cause of disability and death globally. Major risk factors include hypertension and various diseases [57],[58]. Infections, particularly influenza-like illnesses, herpesviruses (CMV, HSV-1/2, varicella, EBV), and HCV, may increase stroke risk through inflammatory mechanisms. The COVID-19 pandemic has further emphasized inflammation's role in cerebrovascular disease, with SARS-CoV-2 potentially inducing strokes via hypercoagulability and immune responses [58]. Vaccination may reduce stroke risk by preventing infections and moderating inflammatory responses when infections occur post-vaccination. However, some studies reported increased stroke rates after influenza, MMR, and diphtheria vaccines. COVID-19 vaccines, upon public rollout, were similarly considered protective against SARS-CoV-2–related strokes [59]. Although rare, some cases of mild transient cerebral ischemia and fatal cerebrovascular events have been reported after COVID-19 vaccination, particularly with adenovirus-based vaccines. Venous sinus thrombosis has been identified as a potentially serious neurological complication associated with these vaccines [60]. Monitoring for signs of cerebral venous thrombosis, such as persistent headache, is recommended in vaccinated individuals. Obstruction in the brain's venous system may lead to elevated intracranial pressure, vessel rupture, and a hypercoagulable state, potentially causing hemorrhagic or ischemic stroke. However, the exact relationship between COVID-19 vaccination and these cerebrovascular events—including effects on blood pressure, platelet levels, and coagulation—remains unclear [61].

The first cases of CVST were linked to AstraZeneca (ChAdOx1 n) and Janssen vaccines. Ahmad et al. reported 11,212 strokes among 4.1 million vaccinated individuals, while Krzywicka et al. identified 213 CVST cases, mostly with AstraZeneca (187) and Pfizer/BioNTech (26) and one with Moderna, in the EudraVigilance database [61]. Reports from France, the US, and Israel showed no increased risk of acute pulmonary embolism, myocardial infarction, or stroke up to 42 days after Pfizer–BioNTech (BNT162b2) vaccination [62]–[64]. Moreover, ischemic stroke due to cerebral artery occlusion occurred at a rate of 0.29–1.76 per million immunizations [65]. A meta-analysis found the highest stroke incidence with AstraZeneca (0.0019%), followed by Janssen (0.0014%) and Pfizer (0.006%) [61]. Another study found no elevated ischemic stroke risk or mortality differences between vaccinated and unvaccinated patients. Vaccine-induced thrombotic thrombocytopenia (VITT) accounted for 3.1% of ischemic strokes. While one study reported increased hemorrhagic stroke risk post-BNT162b2, another found no association [65]. In Mexico, acute stroke was recorded at 0.71 per million doses across six vaccines, including Ad26.COV2-S, BNT162b2, Gam-COVID-Vac, and ChAdOx1 nCov-19 [66].

In Georgia, Nahab et al. found ischemic stroke rates of 8.14, 11.14, and 10.48 per 100,000 for BNT162b2, mRNA-1273, and Ad26.COV2.S, respectively, within 21 days of vaccination. Ad26.COV2.S carried a 57% higher ischemic stroke risk compared to BNT162b2, while no difference existed between mRNA vaccines [67]. Other evidence showed mRNA vaccines such as Moderna and BNT162b2 are associated with a higher rate of arterial problems, mainly due to large artery atherosclerosis (34.9%) [68],[69]. Arterial ischemic events developed with left hemiplegia in a 42-year-old female following bilateral anterior cerebral artery and right middle cerebral artery occlusion, 2 weeks after vaccination with AstraZeneca (ChAdOx1-S) [70]. Elaidouni et al. also reported ischemic stroke after immunization with Sinopharm (BIBP) vaccination in a 36-year-old man [71]. Also, a 51-year-old female complained about right hemiplegia, hemianopia, and global aphasia 7 days after receiving a ChAdOx1-S vaccine [72]. Importantly, studies show that thrombocytopenia and CVST are more likely to follow COVID-19 than vaccination [72],[73].

A meta-analysis indicated a decreased risk of hemorrhagic and ischemic strokes within 28 days after vaccination in cohort studies, while others showed no significant association [74]. mRNA vaccines may activate prothrombotic pathways via cytokine production. The SARS-CoV-2 spike protein could affect BBB integrity, promoting inflammation [72]. Vector-based vaccines may increase platelet factor-4 (PF4) antibodies, triggering coagulation cascades and rare thrombotic events [75]. PF4 triggers a coagulation cascade and results in thrombotic complications. However, lacunar ischemic stroke was reported 10 days after vaccination with Sinopharm (BBIBP-CorV) [76]. Although some associations exist, current evidence indicates that only a very small fraction of stroke cases can be attributed to COVID-19 vaccination, especially considering the vast number of doses administered.

### 5.2. Acute disseminated encephalomyelitis

Acute disseminated encephalomyelitis (ADEM) predominantly affects children, though it can involve adults with varying symptoms. It manifests with impaired consciousness, acute hemiparesis, meningismus, and ataxia. Although the exact pathophysiology of ADEM remains unclear, inflammation in the subarachnoid space and cell-mediated autoimmune responses to myelin proteins—triggered by infection or immunization—are considered key etiological factors. CNS demyelination may occur due to the activation of macrophages, microglia, and Th1 and Th2 cells, which promote the release of immune and inflammatory mediators. Inflammatory cytokines are the main drivers of ADEM. For example, TNF-α and IL-1β are myelinotoxic factors that contribute to the apoptosis of myelin precursor cells, particularly oligodendrocytes. These cytokines can induce the expression of adhesion molecules and HLA-II in CNS vascular endothelial and parenchymal cells, further stimulating microglial activation and demyelination. Elevated levels of IL-2, IL-4, IL-5, IL-10, IFN-γ, TNF-α, and G-CSF have been reported in ADEM, with IL-2 playing a key role by inducing TNF-α and IL-1β production and acting as a myelinotoxic factor through its receptor, IL-2R (CD25) [77]. The subacute onset of neurological complications following viral infection or vaccination can also contribute to the development of ADEM. In the nineteenth century, the first ADEM-like cases were reported after rabies and smallpox vaccinations. Recent research has indicated an association between COVID-19 and ADEM. Fifteen cases of ADEM have been identified following immunization with inactivated vaccines, viral vector vaccines, and mRNA-based vaccines. In five cases, symptoms such as blurred vision and quadriparesis appeared two weeks after the first dose of the SinoVac or AstraZeneca vaccines, leading to ADEM diagnosis. An MRI of a 42-year-old woman, performed 25 days after receiving her initial dose of the AstraZeneca vaccine, identified a substantial, irregular lesion in the right temporal lobe. A subsequent brain biopsy confirmed the diagnosis of tumefactive demyelination [78]. A recent study by the Global Vaccine Data Network reported an increased risk of developing ADEM and transverse myelitis (TM) following the first dose of the AstraZeneca vectored vaccine, particularly within 42 days after adenoviral vaccination, compared to the mRNA-based vaccine (Pfizer-BioNTech) [79]. Additionally, Nabizadeh et al. (2023) reported the occurrence of ADEM following COVID-19 vaccination, analyzing 20 case studies encompassing a total of 54 patients. The findings showed that 45 cases (85.1%) developed ADEM after the first dose, while seven cases (12.9%) were detected after the second dose, with the average onset happening approximately 14 days post-vaccination (ranging from 12 hours to 63 days) [80]. Similarly, Stoien et al. (2023) reported 29 cases of ADEM following SARS-CoV-2 vaccination and 45 cases following COVID-19 [81]. However, some studies found no convincing evidence of a causal link between COVID-19 vaccines and ADEM, indicating the need for further research. Maramattom et al. (2022) similarly found no association between the AstraZeneca vaccine and the incidence of ADEM or encephalitis in the general population [78].

### 5.3. Multiple sclerosis

Multiple sclerosis (MS) is an autoimmune disorder with demyelination and neuroinflammation signs [82]. It is generally categorized into classical, neuromyelitis optica, acute, and concentric sclerosis. Environmental, genetic, and infectious factors can influence its development. While the precise etiology of MS remains unknown, it is widely accepted that inflammation, along with the infiltration of lymphocytes and macrophages into the CNS, plays a crucial role in initiating demyelination and axonal damage. B cells, Th17 cells (via cytokine and chemokine release), and macrophages play critical roles in the pathogenesis and progression of the disease. Cytokines such as IL-1β, IL-4, IL-10, IL-12, IL-17, IL-22, TNF-α, and IFN-γ contribute to MS development through various signaling pathways. Elevated levels of TNF-α, IL-12p40, IL-15, CXCL8 (IL-8), and CXCL13 (B-cell chemokine) have been detected in the CSF and serum of MS patients [61],[83]–[85].

Recent studies have reported cases of MS developing after SARS-CoV-2 vaccinations, raising concerns about a possible link between these vaccines and the onset or relapse of the disease. New-onset or relapsed MS has been observed following the Pfizer, AZD1222, and Sputnik V vaccines. Notably, the Sputnik V vaccine has been associated with MS relapse occurring three days post-vaccination and new-onset MS within a few days, both confirmed by laboratory tests and imaging findings [82]. Khayat-Khoei reported a 24-year-old woman with a history of MS and clinical signs of paresthesia in her right face and upper extremity, along with diplopia, who developed new visual symptoms in her right eye, including blurred vision and pain with eye movement, just one day after receiving the second dose of the Pfizer vaccine. In another case, a woman who completed the full Moderna vaccination series and tested negative for COVID-19 exhibited no significant immediate side effects after the second dose. However, fourteen days later, her MRI and clinical symptoms revealed vision impairment, suggesting the onset of MS [16]. Additionally, they reported that the Moderna vaccine triggered new-onset MS in two cases and exacerbated pre-existing MS symptoms in four cases following the first or second dose of the Pfizer vaccine [16]. Samim et al. (2023) reported a case of a 25-year-old woman with a previously asymptomatic history who developed signs of MS one week after receiving the AstraZeneca vaccine. She met the McDonald criteria (2017) for MS, suggesting that the COVID-19 vaccine may have triggered the symptoms. Additionally, they presented an 8-year-old boy who experienced bilateral visual blurring following Pfizer-BioNTech vaccination [86]. Most studies have not confirmed a causal link between COVID-19 vaccination and MS, despite reports of a possible correlation. However, not vaccinating may raise the risk of infections that could worsen MS.

### 5.4. Transverse myelitis

Transverse myelitis (TM) is a neurological condition marked by inflammation within the spinal cord parenchyma [87]. It is typically caused by direct infection or post-infectious, autoimmune-related inflammation [88]. TM can co-occur with MS, encephalitis, and optic neuritis. Like ADEM, infectious agents in TM can disrupt immune tolerance to self-antigens by activating T lymphocytes, monocytes, and macrophages, leading to the formation of immune complexes and cell-mediated spinal cord injury [89]. Spinal cord injuries induce the release of inflammatory mediators, e.g., IL-6, IL-8, IL-10, matrix metalloproteinase-2 (MMP-2), and MMP-9. Tanaka et al. reported intrathecal upregulation of Th2 cytokines and CCL11 in cases of atopic myelitis [90]. In cases of acute flaccid myelitis, elevated levels of IL-10, interleukin-1 receptor antagonist (IL-1RA), IL-5, IL-6, sCD40L, and IL-7 have been found in CSF, while increased levels of IL-15, IL-5, IFN-γ, MCP-3, IL-12p40, IL-12p70, IL-13, sCD40L, IL-17A, IL-1α, IL-2, IL-7, and TNF-α have been observed in sera [88]–[91].

Clinical trial studies have shown that COVID-19 patients experienced more severe TM manifestations and longer durations of the condition compared to vaccinated individuals, particularly those who received adenoviral vector- and mRNA-based vaccines [88]. In a pre-approval clinical trial for the AstraZeneca vaccine, TM developed 14 days after the second dose. Cases of TM have also been documented following the administration of Moderna, Janssen, and Pfizer-BioNTech's (BNT162b) vaccines. Additionally, other clinical trial findings suggested that the risk of TM was twice as high with the AstraZeneca vaccine [92]. However, another trial did not find a direct association between AstraZeneca (ChAdOx1) and BNT162b2 vaccines and TM [93]. A case of focal myelitis was also reported in Australia following the administration of the AstraZeneca vaccine [94]. Additionally, several voluntary reports submitted to national vigilance boards documented 45 cases of TM linked to the AstraZeneca vaccine and 17 cases to the BioNTech vaccine in the UK, two cases with AstraZeneca and one with BioNTech in Germany, and nine cases in the US, though the specific vaccines involved in the US cases were not identified [95]. Malhotra et al. described a case of TM in a patient who experienced abnormal sensations in both lower limbs eight days after the first dose of the Covishield vaccine in India. Notably, the patient had no prior medical history of this condition [96]. However, the first trial assessing the safety of the AstraZeneca vaccine was temporarily halted after two cases of TM were reported, although no clear evidence could link the vaccine to these cases [97]. Corrêa et al. (2021) reported a case of cervical myelitis after AstraZeneca vaccination [97]. A 64-year-old man with no prior neurological conditions experienced no immediate side effects after receiving the first dose of the Pfizer vaccine. However, eight days later, he developed paresthesia and pain in his upper abdomen, followed by weakness and numbness in his right lower extremity on day 12. His symptoms progressed to involve both lower extremities, along with sphincter dysfunction and balance issues, ultimately leading to a diagnosis of longitudinally extensive TM. Consequently, he chose not to proceed with the vaccination program and did not receive the second dose [97]. Srinivas and colleagues described ATM as a rare complication following COVID-19 vaccination in several cases during acute COVID-19 [98]. Consistently, Wise (2024) recently published findings from the Global Vaccine Data Network cohort, identifying TM and ADEM as very rare side effects of COVID-19 vaccines. The study reported a significant difference in the incidence of TM and ADEM after the first dose of AstraZeneca. The cohort included 99 million vaccinated individuals from eight countries across South America, North America, Oceania, and Europe, with 183,559,462 doses of Pfizer-BioNTech, 36,178,442 doses of Moderna, and 23,093,399 doses of AstraZeneca administered [99]. Morgan et al. (2024) also published data from a self-controlled case series involving 6.7 million individuals in Australia, which demonstrated an increased risk of TM and ADEM following the AstraZeneca vaccine, in contrast to the mRNA-based vaccines [79]. Additionally, Ahmad et al. (2024) conducted a meta-analysis of 21 case series and nine cohort studies, including data on 22,780 participants in COVID-19 vaccination programs. Signs of TM and encephalitis developed 7–11 days after vaccination. The study found that the AstraZeneca vaccine was associated with an increased risk of myelitis, encephalitis, and meningitis [61]. Frontera et al. (2022) also reported a higher risk of TM and ADEM following the Janssen vaccination [15]. However, they emphasized that, due to the rarity of neurological side effects, the wide range of confidence intervals, and the challenge of establishing reliable baseline rates for these conditions, these findings should be interpreted with caution.

### 5.5. Optic neuritis

Optic neuritis (ON) is an acute, demyelinating, or idiopathic optic neuropathy that occurs because of infection, inflammation, or demyelination. It is most commonly linked to MS and is characterized by symptoms such as vision loss, blurred vision, and pain with eye movement [100]. Activated intracerebral T cells, along with systemic T cells that migrate through the blood-brain barrier, and inflammatory mediators such as IL-2, IL-6, and IL-1/3, can contribute to axonal degeneration and neuronal cell death [101],[102]. Cases of ON have been reported following both SARS-CoV-2 infection and COVID-19 vaccination. Studies have documented unilateral or bilateral optic nerve involvement after the first and second doses of vaccines, with onset occurring between 0 and 19 days post-vaccination [65]. Symptoms of ON were observed 19 days after the first dose of the AstraZeneca vaccine (Vaxzevria) in a 42-year-old man (unilateral) and 14 days after in a 24-year-old woman (bilateral) [45]. Also, left ON and sudden vision loss have been reported three weeks after an unspecified COVID-19 vaccination, while symptoms returned to normal following treatment [103]. Additionally, a 33-year-old man experienced unilateral painless blurred vision following the second dose of the Pfizer vaccine. COVID-19 vaccines have also been administered to individuals with a history of demyelinating diseases. For instance, a 48-year-old woman with a history of isolated demyelinating syndrome and a diagnosis of MS developed neurological symptoms, including pain in her right eye during fixation or movement, 15 days after receiving the first vaccine dose. However, inflammation-modifying therapy successfully relieved her eye pain [16]. Furthermore, a case of arthritic anterior ischemic optic neuropathy was reported in a 79-year-old woman who developed ocular pain two days after receiving the second dose of the Pfizer-BioNTech vaccine [104]. Elnahry et al. (2021) reported two cases of optic nerve pathology following AstraZeneca and Pfizer-BioNTech vaccination in individuals with no previous history of symptoms [105]. More recently, in a similar study, Moretti et al. (2024) presented two cases of demyelinating syndromes in a 25-year-old female and an 8-year-old patient, both with previously asymptomatic histories, who developed symptoms 7 and 12 days after receiving the AstraZeneca or Pfizer-BioNTech vaccines, respectively [106]. A case-series study involving data from 5.1 million children in England found no increased risk of adverse conditions within 1–42 days following vaccination with Pfizer-BioNTech, Moderna, or AstraZeneca in children aged 5–11. However, researchers estimated the risk of hospitalization due to demyelinating diseases, particularly optic neuritis, to be approximately 4 per million after the second dose of Pfizer-BioNTech [107]. As a result, there is no conclusive evidence linking vaccination to optic neuritis, and further studies are needed to confirm any potential association.

### 5.6. Encephalitis

Encephalitis is referred to as a condition that leads to inflammation or swelling of the brain, mostly caused by an autoimmune disorder, brain tumors, viral infection, and exposure to toxic substances for a long time [108]. Neuroinflammation in encephalitis can result in encephalopathy by triggering microglial activation and the excessive production of chemokines and cytokines. During viral infection, the expression of MHCI, MHCII, CD40, and CD86 proteins is increased on the surface of microglia. This activation stimulates CD4+ and CD8+ T cells, leading to the excessive production of inflammatory cytokines in the CNS [109],[110]. Alteration of CCL-4, CCL-17, CCL-20, CXCL10, CXCL-13, and TNF-α, IFN-γ, IL-2, IL-4, IL-5, IL-6, IL-9, IL-10, and IL-22 have been reported in the CSF or serum/plasma of patients with encephalitis. Altered homeostasis of CCL2 (MCP-1), CCL3 (MIP-1α), CCL5 (RANTES), CXCL8 (IL-8), sTNFR1, and IL-1β are important for predicting neurological complications [110]. These inflammatory cytokines may reach the brain, activating microglial cells and leading to neuroinflammation. However, potential mechanisms such as molecular mimicry between self-antigens and vaccine antigens or the development of autoimmune responses initiated by vaccines are still being explored.

Encephalopathy has been documented in certain cases following COVID-19 vaccination [111]. Sriwastava et al. documented three cases of encephalitis and encephalopathy occurring after the administration of the first dose of the Moderna vaccine [17]. Another study presented two individuals with no prior history of psychiatric or neurological conditions who developed encephalopathy and seizures with non-convulsive status epilepticus and delirium following the Moderna vaccine [112]. Additionally, in a 64-year-old man, encephalitis symptoms, including drowsiness and fever, appeared ten days after receiving the first dose of the AstraZeneca vaccine, accompanied by hyperintensities in the middle cerebellar peduncle [78]. A recent case series conducted by the Korea Disease Control Agency examined data on meningitis and encephalitis diagnoses from 44,564,345 individuals and 129,956,027 doses of the Pfizer-BioNTech, Moderna, AstraZeneca (ChAdOx1-S), and Janssen (Ad26.COV2.S) vaccines within a 240-day post-vaccination period. The analysis reported 1.9 cases of encephalitis and 3.1 cases of meningitis per 1 million doses. A higher risk of encephalitis within the first 28 days after COVID-19 vaccination was found, particularly following the AstraZeneca (ChAdOx1-S) vaccine. However, authors highlighted that the absolute risk remained low and should not undermine public confidence in COVID-19 vaccines [113]. Lopez-Gonzalez et al. (2024) described a 36-year-old woman suspected of having autoimmune encephalitis, who experienced progressive behavioral and cognitive changes following the administration of the second dose of the Moderna vaccine [114]. In this regard, several studies have also reported cases of acute encephalitis or autoimmune encephalitis following the first or second dose of various COVID-19 vaccines [115]–[118]; however, these studies could not confirm the incidence of encephalitis or establish a causal link between COVID-19 vaccination and the development of encephalitis.

### 5.7. Bell's palsy

Bell's palsy (BP), or idiopathic facial paralysis, is mostly referred to as acute unilateral peripheral facial nerve palsy [119]. The BP occurs spontaneously but may also occur following other complications such as the herpes simplex virus infection [120]. Some vaccines, such as HIV, hepatitis B, Epstein–Barr virus (EBV) [121], influenza, and meningococcal conjugate vaccines, have also been involved in BP development [122],[123]. Immune-mediated mechanisms targeting basic myelin protein have been proposed as contributing factors in the pathogenesis of BP. The role of CD8+ T cells and neutralizing antibodies has been established in viral infections [124]. It has been suggested that CD8+ T cells are crucial for perforin- and IFN-γ-dependent mechanisms that help maintain viral latency. Sawy et al. (2012) found a decrease in CD4+ T helper cells while observing a significant increase in B lymphocytes during the acute phase of BP [124].

Following the COVID-19 pandemic, some patients were referred to medical centers with symptoms of BP [125],[126], which may occur by stimulation of the immune response through molecular mimicry with the host's self-antigens or virus activity per se. The virus spike glycoprotein may cross-react with the host's proteins and trigger inflammatory responses and BP symptoms in susceptible COVID-19 patients [127]. Ozonoff et al. (2021) reported a rate of BP 3.5–7 times higher than expected in the general population in the combined phase 3 of Pfizer-BioNTech and Moderna trials [128]. In addition to Moderna [129] and Pfizer-BioNTech [130], BP has also been reported following Janssen, SinoVac [119], and AstraZeneca [131] vaccines in a case series study. The timing of BP outbreaks after vaccination is uncertain, although it has been suggested to happen about four weeks after vaccination [59]. A recent multi-country cohort study on 99,068,901 vaccinated people with 183,559,462 doses of Pfizer-BioNTech, 36,178,442 doses of Moderna, and 23,093,399 doses of AstraZeneca (ChAdOx1-S) vaccines found that the number of patients diagnosed with BP increased following the first dose of Moderna and Pfizer-BioNTech (100). Moreover, Ahmad et al. (2023) reported that the most frequent neurological problems following vaccinations were stroke (4151 cases), BP (3785 cases), seizures (1977 cases), GBS (645 cases), and CNS infections (469 cases) [61]. Although BP is a rare neurological complication associated with COVID-19 vaccines, current evidence suggests that the risk of developing BP is 2–3 times higher with mRNA vaccines compared to traditional vaccines. Therefore, this risk should be considered in individuals with a history of BP. However, this limitation should not hinder the COVID-19 vaccination program. The risk-benefit ratio must be assessed to recommend alternative safe vaccines or to conduct more studies for reliable results [131].

### 5.8. Guillain–Barre syndrome

Guillain–Barré syndrome (GBS) is an autoimmune disorder characterized by progressive demyelination and motor dysfunction, typically beginning in the distal extremities and potentially involving the arms, legs, and, in some cases, respiratory muscles. GBS is generally categorized into two subtypes: the axonal subtype (which includes motor or motor and sensory axonal neuropathy) and the demyelination subtype (inflammatory demyelinating polyneuropathy) [132],[133]. Several factors contribute to the etiology of GBS, including host susceptibilities and immune homeostasis. Similar to BP, the majority of patients experience a viral or bacterial infection before the onset of neurological symptoms, which is linked to an increase in both cellular and humoral immune responses [134]. Pathogenic agents play a crucial role in initiating a systemic immune response targeting the nerves or nerve roots in the peripheral nervous system, which triggers cross-reactive cellular and humoral immune activities, leading to demyelination, axonal injury, or both, along with other GBS symptoms [133]. It is generally proposed that macrophages, B cells, T cells, and the complement system initiate and execute attacks on axons and Schwann cells. In GBS, various T-cell subsets, including CD3+, CD4+ (CD4+ CD25+), CD8+, CD16+, CD95+ (Fas), and γδ T cells, along with CD5+ and memory B cells, are involved in modulating both inflammatory and anti-inflammatory responses [135]–[137].

Recently, various studies have also reported an increase in inflammatory cytokines, such as TNF-α, IFN-I, IFN-γ, IL-1β, IL-4, IL-6, IL-8, IL-12, IL-16, IL-17, IL-18, IL-22, IL-23, and IL-27, and chemokines, such as MCP-1, MIP-1a, CCR2, RANTES, IP-10, and CXCL10, in GBS patients' serum/CSF [138]–[141]. Notably, IL-1β, TNF-α, and IL-6 serve dual roles, exhibiting both pro-inflammatory and protective effects in GBS. The protective effects of IL-6 are linked to its ability to reduce IL-1β and TNF-α levels and promote remyelination. Meanwhile, cytokines such as IL-4, IL-10, IL-17, IL-21, IL-27, IL-35, IL-37, and TGF-β serve anti-inflammatory roles and help suppress the disease [138]–[140],[142]. The main effect of IL-10 is macrophage inhibition, together with TNF-α and IL-1 production; Myhr et al. (2003) proposed that IL-10 and its promoter polymorphisms can develop GBS by increasing the ganglioside antibody generation [143]. Moreover, IL-23, a recently identified member of the IL-12 family, primarily targets memory T cells and plays a crucial role in mediating cellular immune responses [142].

Recent studies have demonstrated an increase in cases of GBS during the COVID-19 pandemic and after COVID-19 vaccination. While GBS has been suggested to be the most common peripheral nervous system issue among vaccine-related neurological complications (accounting for 67%), there is no conclusive evidence linking COVID-19 vaccination to an increased risk of GBS or its associated complications [17]. Nagdev et al. reported a 23-year-old man with numbness and quadriparesis (numbness in the trunk, hands, and lower limbs) on both sides, consistent with GBS. This incident occurred ten days after the individual received the first dose of the AstraZeneca vaccine, with MRI scans of the brain and spinal cord revealing evidence of nonspecific central demyelination. He had no prior history of demyelinating disease [144]. It is estimated that 38 new cases of GBS occur per 10 million individuals following administration of the AstraZeneca vaccine, equating to 227 cases per 51 million doses administered [99]. In comparison, 145 new cases of GBS have been reported per 10 million individuals who tested positive for COVID-19 [145]. The GBS frequency was predicted to be 1.4–10-fold more than estimated in the same population and period of time [146]. Another study revealed that the rate of GBS was 8–19 cases per million per year in adults. Consequently, the first and second doses of COVID-19 vaccines were associated with an estimated 900–2200 and 1500–3700 GBS cases, respectively, within six and ten weeks after vaccination. Additionally, a variant of GBS, known as Miller–Fisher syndrome, was diagnosed in a 37-year-old man following the first dose of the Pfizer-BioNTech vaccine [45]. However, Taga et al. reported that mRNA vaccine was not associated with GBS [146]. A trial reported that 99 cases out of 622 individuals with GBS had a positive COVID-19 test before vaccination. Recent evidence showed GBS symptoms were observed eleven days after the initial features of SARS-CoV-2 infection detection, two weeks after the first dose of Pfizer-BioNTech vaccine in a woman [17], and four days after the second dose of Pfizer-BioNTech in a man [45]. In addition, Patone et al. reported that the first dose of AstraZeneca (ChAdOx1n) vaccine was associated with a higher risk of GBS manifestation or death after 15–21 days and 22–28 days, unlike Pfizer-BioNTech vaccine [19]. A similar report from a Mexican cohort study followed 3,890,250 individuals within 30 days of the first Pfizer vaccination and identified seven classic GBS cases (6 days following vaccination), with an observed incidence of 0.18/100,000; among the 613,780 cases, no GBS symptoms were found after receiving both doses. Authors demonstrated no association between COVID-19 vaccination and GBS incidence [147]. Researchers have proposed that the occurrence of GBS following COVID-19 vaccination, particularly with mRNA-based vaccines, is less common. They suggest that molecular mimicry involving neural components may be associated with the structure of adenoviral vectors, which could explain the safety profile of mRNA vaccines. Additionally, they reported that among AstraZeneca, Pfizer, and Moderna vaccines, vaccination with AstraZeneca increased the number of GBS admissions by 2.6-fold when compared to a similar period over the past three years [146]. GBS was presented four weeks after receiving the first dose of the AstraZeneca vaccine in 16 cases, and Pfizer and Moderna in one case. After AstraZeneca vaccination, the GBS variant was associated with bifacial weakness and distal paresthesia. In another preliminary record on 13.2 million individuals receiving the Janssen (Ad26.COV2.S) vaccine, 132 cases reported GBS, with a predicted rate of 9.8 cases/million doses [148]. Overall, GBS was reported in persons exposed to AstraZeneca [149], Moderna [150], Pfizer-BioNTech, and AZD1222 vaccines [132]. Besides, Sriwastava et al. (2022) reported that GBS admission following the first dose of AstraZeneca, the second dose of the Pfizer-BioNTech, and a single dose of the Janssen vaccine, was 64%, 22%, and 14%, respectively, unlike COVAXIN, SinoVac, and Sputnik vaccines [17]. The estimated rate of neurological implications after SARS-CoV-2 infection is up to 617-fold higher than rates after receiving COVID-19 vaccination, proposing that the overall advantages of COVID-19 vaccination outweigh the risks [15].

## 6. Conclusions

Vaccination against SARS-CoV-2 remains the most beneficial way to control severe illness and the pandemic; however, concerns regarding potential neurological complications following vaccination persist in the scientific literature and society. Stroke, ADEM, TM, MS, BP, GBS, encephalitis, and ON have been reported following COVID-19 vaccines, especially AstraZeneca, Janssen, Pfizer, and Moderna. Although small case series and isolated reports have raised concerns about the causality between vaccines and these adverse events, there is no conclusive evidence that vaccines are the direct cause. Nevertheless, the causal relationship remains to be elucidated due to the lack of a consistent pattern regarding the type of vaccine or the timing of symptoms after vaccination. In contrast, most neurological complications may be coincidental or related to pre-existing conditions. Additionally, studies indicate that the risk of neurological complications following SARS-CoV-2 infection itself is far greater than the risk posed by vaccination. Given that billions of people have been vaccinated against COVID-19 worldwide, serious neurological side effects are exceptionally uncommon. Data from large epidemiological studies and global surveillance centers consistently demonstrate that these adverse events are very rare, reinforcing the strong overall safety profile of the vaccines. However, large-scale prospective studies and long-term monitoring are essential to fully address the relationship between vaccines and neurological side effects. Further research may also guide the development of safer vaccines and help refine recommendations for individuals at risk of these complications.

## Use of AI tools declaration

The authors declare they have not used Artificial Intelligence (AI) tools in the creation of this article.


