# Supplementary material for: COVID-19 vaccines and neurological disorders: A narrative review of immune responses and adverse reactions
Source: AIMS Neurosci. 2025 Jun 18;12(2):222–49. doi: 10.3934/Neuroscience.2025013 (PMC12287643; doi:10.3934/Neuroscience.2025013)
Supplement: Supplementary file 1 [file neurosci-12-02-013-s001.pdf]

AIMS Neuroscience

AIMS Neuroscience, 12(2): 222–249.
DOI: 10.3934/Neuroscience.2025013
Received: 17 January 2025
Revised: 27 May 2025
Accepted: 30 May 2025
Published: 18 June 2025

https://www.aimspress.com/journal/neuroscience

Review

COVID-19 vaccines and neurological disorders: A narrative review of immune responses and adverse reactions

Mehran Joodaki[1], Farhad Seif[2], Azadeh Afzalnia[3], Nikoo Emtiazi[4], Mona Merati Shirazi[5], Behnaz Ashtari[6,7], Seyed Mohamad Hosseinian[8,*] and Nasrin Hosseini[9,*]

1 Shariati Hospital, Tehran University of Medical Sciences, Tehran, Iran
2 Department of Photodynamic, Medical Laser Research Center, Yara Institute, Academic Center for Education, Culture, and Research (ACECR), Tehran, Iran
3 Rajaei Cardiovascular Medical and Research Center, Iran University of Medical Sciences, Tehran, Iran
4 Department of Pathology Medicine, Rasool Akram Hospital, School of Medicine, Iran University of Medical Sciences, Tehran, Iran
5 Biomedical Engineering Faculty, Biomechanics Department, Islamic Azad University, Science and Research Branch, Tehran, Iran
6 Radiation Biology Research Center, Iran University of Medical Sciences, Tehran, Iran
7 Department of Medical Nanotechnology, Faculty of Advanced Technologies in Medicine, Iran University of Medical Sciences, Tehran, Iran
8 Rheumatology Department, Shafa Hospital, Kerman University of Medical Sciences, Kerman, Iran
9 Neuroscience Research Center, Iran University of Medical Sciences, Tehran, Iran

* **Correspondence:** Email: Nasrin Hosseini: nasrinhosseini501@yahoo.com; Seyed Mohamad Hosseinian: Mohamadhosseinian@gmail.com.

Supplementary

Table 1. Different platforms of COVID-19 vaccines and vaccinations.

Vaccine platform	Vaccine type	A	N
PS	rS/Matrix M1-Adjuvant (Full length recombinant)/Glycoprotein nanoparticle vaccine adjuvanted with Matrix M) NVX-CoV2373/CovovaxTM/NVX-CoV2515 (booster) or Novavax	IM	2
	COVAX-19® Recombinant spike protein + adjuvant/ SPIKOGEN®	IM	2
	Razi Cov Pars, recombinant spike protein	IM and IN	3
VVnr	ChAdOx1-S - (AZD1222), Covishield, Vaxzevria	IM	1–2
	Recombinant COVID-19 vaccine (adenovirus type 5 vector) for Inhalation (Ad5-nCoV-IH)	IH	1
	Gam-COVID-Vac Adeno-based (rAd26-S+rAd5-S)<br>- Sputnik V COVID-19 vaccine<br>- Sputnik-Light Vector Vaccine (rAd26 platform	IM	2
	Ad26.COV2.S	IM	1–2
	iNCOVACC (BBV154), Adenoviral vector COVID-19 vaccine	IN	1
	AZD2816; adenoviral vector ChAdOx platform and based on the Beta (B.1.351) variant	IM	2
VVr + APC	Covid-19/aAPC vaccine. The Covid-19/aAPC vaccine is prepared by applying lentivirus modification with immune modulatory genes and the viral minigenes to the artificial antigen presenting cells (aAPCs).	SC	3
	Dendritic cell vaccine AV-COVID-19 (consisting of autologous dendritic cells loaded with antigens from SARS-CoV-2, with or without GM-CSF)	IM	1
DNA-BV	INO-4800 + electroporation	ID	2
	nCov vaccine	ID	3
	CORVax12 - Spike (S) Protein Plasmid DNA Vaccine	ID	2
	COVIGEN	ID/IM	2
Inactivate Virus	CoronaVac; inactivated SARS-CoV-2 vaccine (vero cell)	IM	2
	Inactivated SARS-CoV-2 vaccine (Vero cell) WIBP COVID-19 vaccine	IM	2
	Inactivated SARS-CoV-2 vaccine (Vero cell), vaccine name BBIBP-CorV	IM	2
	SARS-CoV-2 vaccine (vero cells)	IM	2
	QazCovid-in® - COVID-19 inactivated vaccine	IM	2
	BBV152 vaccine	IM	2
	COVAXIN	IM	2
	Covi Vax, inactivated coronavirus vaccine	IM	2
	Osvid-19 inactivated vaccine for Covid-19	IM	2
	Omicron COVID-19 inactivated Vaccine (Vero Cell)	IM	2

Continued on next page

Vaccine platform	Vaccine type	A	N
RNA-BV	mRNA-1273	IM	2
	Spikevax	IM	2
	BNT162b2 (3 LNP-mRNAs), also known as "Comirnaty")	IM	2
	CVnCoV Vaccine	IM	2
	SARS-CoV-2 mRNA vaccine (ARCoV)	IM	2
	RNA-1273.351/A lipid nanoparticle (LNP)-encapsulated mRNA-based vaccine that encodes for a full-length, prefusion stabilized S protein of the SARS-CoV-2 B.1.351 variant.	IM	3
	mRNA-1283 /mRNA-1283.211	IM	2
	mRNA COVID-19 vaccine	IM	2
	mRNA-1273.211. A multivalent booster candidate combining mRNA-1273 plus mRNA-1273.351.	IM	1
	mRNA-1273.529 - Booster	IM	1
VVr	V591-001- Measles-vector based (TMV-o38)	IM	1–2
	rVSV-SARS-CoV-2-S Vaccine (IIBR-100)	IM	1
	COVIVAC. Newcastle Disease Virus (NDV) expressing membrane-anchored pre-fusion-stabilized trimeric SARS-CoV-2 S protein +/- adjuvant CpG 1018	IM	2
	NDV-HXP-S; A Live Recombinant Newcastle Disease Virus-vectored COVID-19 Vaccine	IM/IN	1
VLP	RBD SARS-CoV-2 HBsAg VLP vaccine.	IM	2
	Coronavirus-Like Particle COVID-19 (CoVLP)/ MT-2766/Other Name: CoVLP, AS03 adjuvant	IM	2
	VBI-2902a. An enveloped virus-like particle (eVLP) of SARS-CoV-2 spike (S) glycoprotein and aluminum phosphate adjuvant.	IM	2
	SARS-CoV-2 VLP Vaccine/Vaccine-Wuhan; Vaccine-Alpha variant; Vaccine-Wuhan+Alpha variant	SC	2
	ABNCoV2 capsid virus-like particle (cVLP) +/- adjuvant MF59	IM	2
	SARS-CoV-2 Vaccine LYB001, a receptor-binding domain (RBD) from SARS-CoV-2 and virus-like particle (VLP) vector, adjuvanted with aluminum hydroxide	IM	3
VVr + APC	Covid-19/aAPC vaccine. The Covid-19/aAPC vaccine is prepared by applying lentivirus modification with immune modulatory genes and the viral minigenes to the artificial antigen presenting cells (aAPCs).	SC	3
	Dendritic cell vaccine AV-COVID-19. A vaccine consisting of autologous dendritic cells loaded with antigens from SARS-CoV-2, with or without GM-CSF	IM	1
LAV	COVI-VAC	IN	1–2
	MV-014-212, a live attenuated vaccine that expresses the spike (S) protein of SARS-CoV-2	IN	1

Continued on next page

Vaccine platform	Vaccine type	A	N
VVnr + APC	LV-SMENP-DC vaccine. Dendritic cells are modified with lentivirus vectors expressing Covid-19 minigene SMENP and immune modulatory genes. CTLs are activated by LV-DC presenting Covid-19 specific antigens	SC and IV	1
Bac Ag-Sp-Vec	COVID19 Oral Vaccine Consisting of Bacillus Subtilis Spores	Oral	3

Note: Protein subunit (PS), Live Attenuated Virus (LAV), Virus Like Particle (VLP), Viral Vector Non-replicating (VVnr), Viral vector replicating + Antigen Presenting Cell (VVr+APC), DNA based vaccine (DNA-BV), RNA based vaccine (RNA-BV), Viral Vector replicating (VVr), Viral vector non-replicating + Antigen Presenting Cell (VVnr+APC), Bacterial antigen-spore expression vector (Bac Ag-Sp-Vec), Not reported (NR), Intramuscular Route (IM), Subcutaneous Route (SC), Intranasal Route (IN), Intra dermal (ID), Inhaled (IH), Aerosol (AE), Number of doses (N), Administration route (A).

AIMS Press

© 2025 the Author(s), licensee AIMS Press. This is an open access article distributed under the terms of the Creative Commons Attribution License (https://creativecommons.org/licenses/by/4.0)
